# Antibacterial Activity of Nanoparticles

**DOI:** 10.3390/nano11061391

**Published:** 2021-05-25

**Authors:** Vi Khanh Truong, Nghia Phuoc Truong, Scott A. Rice

**Affiliations:** 1 School of Science, RMIT University, 124 La Trobe Street, Melbourne, VIC 3000, Australia; 2 Monash Institute of Pharmaceutical Sciences, Monash University, Melbourne, VIC 3052, Australia; nghia.truong@monash.edu; 3 The Singapore Centre for Life Sciences Engineering and the School of Biological Sciences, College of Science, Nanyang Technological University, 50 Nanyang Avenue, Singapore 639798, Singapore; rscott@ntu.edu.sg; 4 ithree Institute, The University of Technology Sydney, Ultimo, NSW 2007, Australia

Antimicrobial resistance (AMR) is predicted to soon become one of the most serious threats to human and animal health [1,2]. It can result in extended hospital stays, increased medical costs, and even death. Both the prevalence of AMR and the rise in biofilm-associated infections are increasing the demand for new and reliable treatments for these infections. Nanotechnology offers a revolutionary new solution to this challenge. This analysis focuses on the most recent advancements in the field of antimicrobial, inorganic-based nanomaterials, and their action against bacteria and bacterial biofilms. In this Special Issue, nanomaterials that prevent bacterial adhesion and nanomaterials that treat infections once they have developed are described. To combat infection, nanoparticles with inherent antibacterial activity and nanoparticles acting as nanovehicles are identified, with a focus on the design of carrier nanosystems with properties that target bacteria and biofilm. 

In short, this Special Issue includes three reviews and twelve research papers, where they are grouped based on their functionality. These reviews summarise the current state of knowledge about the antimicrobial properties of ZnO and Ag nanoparticles towards bacteria and viruses [3,4]. In particular, Lamouroux et al. discuss the limitations of Ag nanoparticles, including facts and opinions about their antibacterial results [5]. This Special Issue will highlight cutting-edge research on antibacterial nanomaterials used in a variety of applications. Additionally, the Special Issue will illustrate the difficulties and challenges associated with developing successful antimicrobial nanomaterials.

Some of the articles focus on sustainable production, such as Ag nanoparticles that were synthesised using *Areca catechu* and *Benincasa hispida* [6,7]. Additionally, Ag nanoparticles were found to inhibit *Staphylococcus epidermidis* biofilm development [8]. Additionally, it was discovered that other Ag nanoparticles synthesised from *Phyllanthus urinaria*, *Pouzolzia zeylanica*, and *Scoparia dulcis* leaf extracts inhibited the growth of *Aspergillus niger*, *Aspergillus flavus*, and *Fusarium oxysporum* [9]. Another nanoparticle, cerium oxide, inhibited the growth of *Escherichia coli*, *Salmonella typhimurium*, *Listeria monocytogenes*, *Staphylococcus aureus*, and *Bacillus cereus* effectively [10]. Biogenic silica (b-SiO_2_) nanopowders from rice husk ash were prepared by chemical method [11]. This b-SiO_2_ nanopowder exhibited antibacterial activity against *Staphylococcus aureus* and *Escherichia coli*. Surfactin-loaded κ-carrageenan oligosaccharides linked cellulose nanofibers (CO-CNF) nanoparticles were developed to inhibit the growth of *Fusobacterium nucleatum* and *Pseudomonas aeruginosa* [12]. Also, these CO-CNF nanoparticles were found to be anti-inflammatory. 

Other research has resulted in the development of nanocomposites, such as Zn-doped phosphate-based glass and ZnMn_2_O_4_–Chitosan [13,14]. Gram-positive and Gram-negative pathogens were eradicated using these nanocomposites. 

Some studies in this Special Issue developed functionalized quantum dots, including titanium–gadolinium quantum dots, peptide-conjugated carbon dots, and CdSe quantum dots [15,16,17]. These nanomaterials were discovered to be highly effective at eliminating Gram-positive and Gram-negative bacteria. Additionally, some of these molecules have been determined to be biocompatible.

In summary, this Special Issue contains a collection of papers that include an overview of the various solutions based on two distinct approaches: preventing infection through surface modification of nanoparticles and combating infection through precise design of nanoparticles with implicit antimicrobial activities or nanoparticles as carriers of antibacterial drugs. These studies suggest developing novel and practical solutions to prevent pathogenic microorganisms from spreading from the surface of biomedical instruments to the surrounding hospital and other environments. Nanotechnology proves to be an excellent tool in the fight against infection when used for this purpose. One of the more recent efforts has been the discovery of antimicrobial nanomaterials, which can prevent pathogens from developing resistance. To date, most of these studies have evaluated their in vitro cytotoxicity using various cell cultures. However, for such approaches to move from the laboratory towards clinical implementation, in vivo models of infection are needed to gain a better understanding of the biological effects of these approaches, including toxicity, biodistribution, resistance development, and mechanism of action.
